# An Elongator mouse model of ALS spotlights TDP-43 in the motor neuron nucleolus

**DOI:** 10.1038/s42003-025-08701-9

**Published:** 2025-08-21

**Authors:** Magge Snow, BreAnna Cameron, Renzie Pond, Rachel Trudell, Sara Snyder, Lauryn Torres-Hernandez, Devyn Deschamps, Danara Tulimaiau, Kiana Hawkinson, Morgan Russell, Danielle Horan, Joseph Walters, James H. Fox, Britni Arlian, Alain Chariot, Laurent Nguyen, Lynn George

**Affiliations:** 1 Department of Biological and Physical Sciences, Montana State University Billings, Billings, MT USA; 2 College of Veterinary Medicine, Washington State University, Pullman, WA USA; 3 Department of Physiology, Johns Hopkins University School of Medicine, Baltimore, MD USA; 4 Department of Neuroscience, Duke University, Durham, NC USA; 5 Department of Chemistry and Biochemistry, Montana State University, Bozeman, MT USA; 6 Billings Clinic Department of Psychiatry, Billings Clinic, Billings, MT USA; 7 Animal Resources Center, Montana State University, Bozeman, MT USA; 8 Laboratory of Cancer Biology, Interdisciplinary GIGA Institute, University of Liège, Liège, Belgium; 9 WELBIO Department, WEL Research Institute, Wavre, Belgium; 10 Laboratory of Molecular Regulation of Neurogenesis, GIGA Institute, University of Liège, Liège, Belgium

**Keywords:** Amyotrophic lateral sclerosis, Cellular neuroscience

## Abstract

Dysfunction of Elongator is associated with amyotrophic lateral sclerosis (ALS). Here, we describe mouse models in which either Elongator subunit 1(*Elp1*) or subunit 3 (*Elp3*) is selectively ablated in alpha motor neurons of the spinal cord. These mice exhibit a progressive loss of motor strength and motor neuron degeneration. To interrogate the molecular mechanisms that contribute to motor neuron cell death in these mice, we examine multiple disease pathways, including the expression of TDP-43 whose cytoplasmic aggregation is associated with the human disease. Although TDP-43 is a well-characterized nuclear protein functioning in RNA metabolism and gene transcription, here we document TDP-43’s robust presence in the nucleolus of wild-type motor neurons and its clearance from both the nucleus and the nucleolus of motor neurons in *Elp* conditional knockout mice. Thus, this study directly links dysfunction of Elongator with nucleolar disruption and TDP-43 clearing, two hallmark cellular pathologies of ALS.

## Abstract (web summary)

Selective ablation of Elongator in a conditional knockout mouse model of amyotrophic lateral sclerosis leads to motor neuron degeneration and reduced levels of nuclear and nucleolar TDP-43

## Introduction

ALS is a relentlessly progressive neurological disease that ravages motor neurons and the voluntary muscles they innervate^1–3^. Interestingly, ALS shares clinical and pathological features with a form of dementia known as frontotemporal dementia (FTD), a common form of dementia second only to Alzheimer’s in incidence^4,5^. Recently, several genes linked to ALS have been shown to also cause FTD, such that the two conditions are now considered to be at opposite ends of the same disease spectrum^6,7^. Despite its prevalence and concentrated efforts at finding a cure, there are currently no effective treatments for ALS. Once diagnosed, patients typically succumb to the disease within five years^8^.

Although ALS can result from inherited mutations in a diverse set of genes, approximately 90% of cases are sporadic^9,10^. A pathological feature of disease present in 97% of ALS and FTD cases, including both inherited and sporadic forms, are cytoplasmic aggregates containing the protein TDP-43^4,8,11,12^. It remains unclear whether these aggregates are themselves toxic to the cell, or whether in forming aggregates an essential function of TDP-43 is lost. TDP-43 is a highly conserved and ubiquitously expressed 43 kDa protein involved in several RNA processing events, including splicing, translation, and degradation^13,14^. Targeted depletion of the gene encoding TDP-43 (*TARDBP)* in mice leads to motor neuron degeneration and a phenotype similar to ALS^15^. This result suggests that loss of TDP-43’s normal function in the cell may in fact be the underlying pathophysiologic mechanism driving disease. Separate from TDP-43, recent studies show that stress and dysfunction of the nucleolus, the site of ribosome biogenesis, is an early, pathological feature common to both familial and sporadic forms of ALS^16–21^.

Dysfunction of Elongator, a large, six-subunit complex encoded by genes *ELP1-ELP6* can also contribute to ALS. Elongator mediates the addition of mcm^5^ to tRNAU_34_, a chemical modification essential to the translational efficiency of transcripts that preferentially use either AA- or AG- ending codons for lysine, glutamine, and glutamic acid. In a microsatellite association study of 1483 individuals (781 ALS patients and 702 controls) from three different countries (U.S.A., Belgium, and the UK), allelic variants of *Elp3* were associated with ALS (*P* = 1.96 × 10^−9^) and were shown to correspond to a 59% reduction in ELP3 protein levels in the motor cortex of ALS patients compared to controls^22^. In an additional study of genes that modify the course of disease in individuals carrying repeat expansions of *C9ORF72*, the most common cause of familial ALS, single nucleotide polymorphisms (SNPs) in *ELP3* reduced the percentage of patients alive eight years after disease onset from ~58% in controls, to ~12% in patients carrying a minor *ELP3* SNP^23^. A third study showed that *Elp3* expression is reduced in the brains and spinal cords of SOD1^G93A^ mice that model *SOD1* related familial forms of ALS and that exogenous overexpression of *Elp3* attenuates the ALS-like phenotype of these mice^24^. Given this precedent, we sought to generate an Elongator knockout mouse model that would be useful for interrogating the molecular and cellular mechanisms via which Elongator dysfunction may contribute to motor neuron disease. To this end, we selectively ablated either *Elp1* or *Elp3* in alpha motor neurons of the spinal cord. In investigating the pathways that go awry in these mice and ultimately culminate in neurodegeneration, we document TDP-43’s presence in the neuron nucleolus in wild-type mice, and its clearing from both the nucleus and the nucleolus in Elongator conditional knockout (CKO) mice. Thus, this study connects dysfunction of Elongator with two hallmark cellular pathologies of ALS; nucleolar disruption and TDP-43 clearing.

## Results

### Elongator subunits are expressed in spinal cord motor neurons

**Fig. 1:**
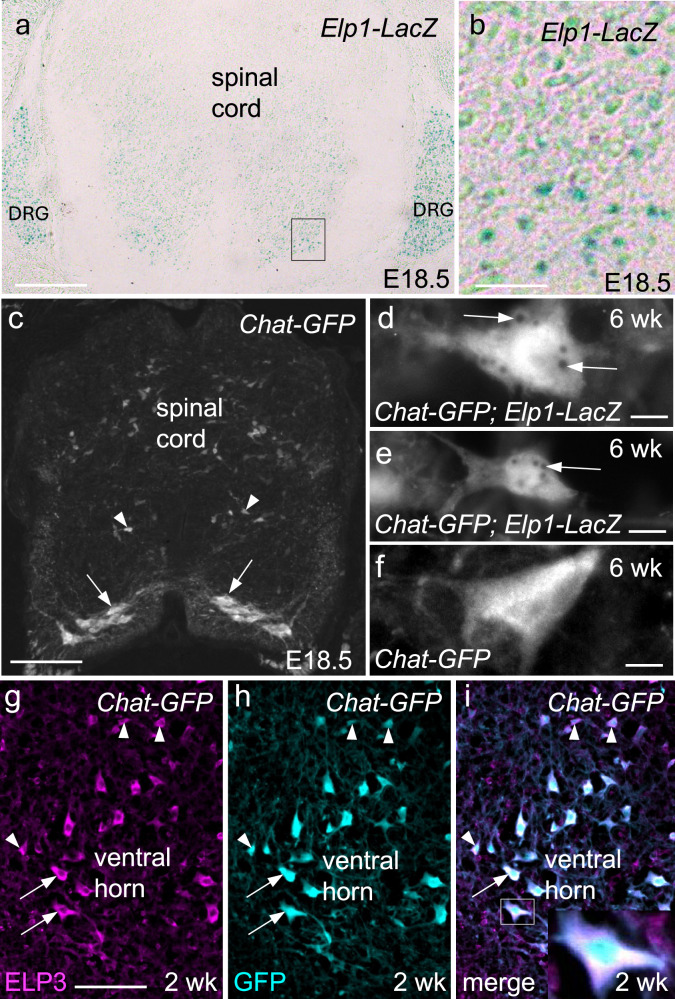
Expression of Elongator subunits in spinal cord motor neurons. **a**–**c** E18.5. **a**, **b** An *Elp1-LacZ* reporter construct shows robust activity in the ventral horn of the spinal cord where alpha motor neurons are located. **b** Enlargement of the boxed region in (**a**). **c** In *Chat-GFP* embryos, GFP is present in both large diameter ventral horn neurons (≥ 440 μm^2^, likely alpha motor neurons, arrows) and in smaller motor neurons (likely gamma motor neurons, arrowheads). **d**–**f** Adult (6 weeks). **d**, **e** Combination of *Elp1-LacZ* (black puncta) and *Chat-GFP* indicate that *Elp1* is expressed in both large (**d**) and small (**e**) motor neurons. **f** Negative control. No puncta are present in motor neurons from *Chat-GFP* mice that lack the *Elp1-LacZ* cassette when incubated with x-gal substrate. **g**–**i** Spinal cord sections from 2-week-old *Chat-GFP* mice. IF using an anti-ELP3 antibody in *Chat-GFP* mice demonstrates that ELP3 is present in both large (arrows) and small (arrowheads) motor neurons. **i** Inset shows an enlargement of the boxed large diameter motor neuron. DRG dorsal root ganglion. Scale bar = 150 μm in (**a**), 75 μm in (**c**), 7.5 μm in (**d**–**f**), 100 μm in (**g**–**i**).

To explore Elongator’s potential contribution to motor neuron disease, we first wanted to confirm that spinal cord motor neurons express Elongator subunits. In the absence of an ELP1 antibody that works for immunofluorescence (IF) in mouse, we combined an *Elp1-LacZ* reporter (Fig. 1a, b), and a *Chat-GFP* reporter (Fig. 1c). Analysis of spinal cord sections from adult mice expressing both constructs shows that large motor neurons (≥ 440 μm^2^) in the spinal cord ventral horn that are most likely alpha motor neurons, and smaller GFP-positive neurons, are both sites of *Elp1* expression (Fig. 1d–f). To determine whether *Elp3* is expressed in motor neurons, a mouse anti-ELP3 antibody was used in combination with the *Chat-GFP* reporter. Fig. 1g–i show that like *Elp1, Elp3* is also expressed in both large and small motor neurons. These results indicate that dysfunction of the Elongator complex could directly affect spinal cord motor neurons.

### Elongator conditional knockout mice exhibit a progressive motor phenotype

**Fig. 2:**
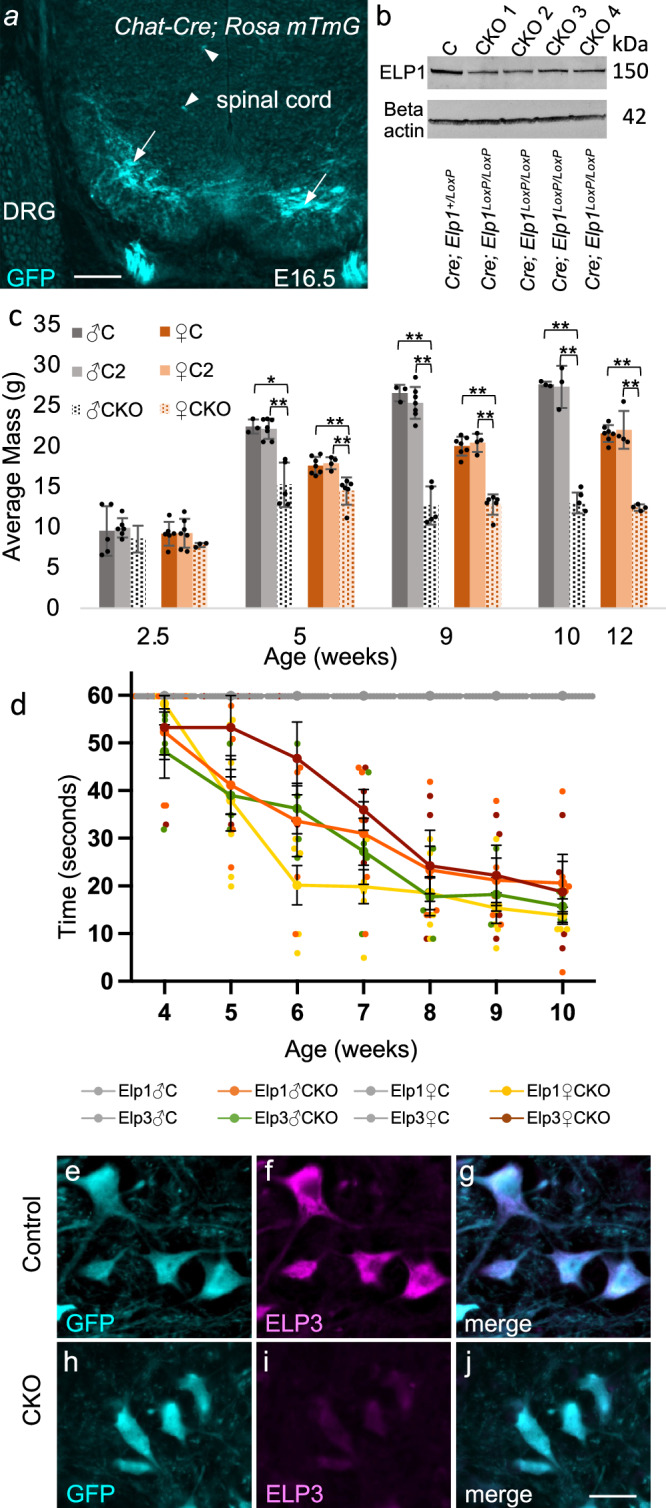
Generation of cholinergic neuron-specific Elongator CKO mice. **a** E16.5. In *Rosa mTmG* mice, GFP is produced in cell types expressing Cre recombinase. In *Chat-Cre; Rosa mTmG* mice, robust GFP expression is seen in large motor neurons in the ventral horn (arrows) and only sporadically seen in smaller motor neurons (arrowheads). **b** ELP1 protein levels are reduced in spinal cords of *Elp1* CKO mice. Residual ELP1 in the CKO lanes corresponds to intact *Elp1* expression in spinal cord cell types other than motor neurons. For uncropped images and size standards, see Fig. S2. **c** Masses of control and *Elp1* CKO mice. No male data are included in the 12-week timepoint since males reach a humane endpoint at 10 weeks. Control 1, C (*Chat-Cre; Elp1*^*+/LoxP*^); Control 2, C2 (*Elp1*^*LoxP/LoxP*^). *P < 0.001, **P < 0.0001 (one-way ANOVA, Tukey’s HSD test). Bars = SD. For source data see Supplementary Data 1. **d** PaGE testing. Both male and female *Elp1* and *Elp3* CKOs begin to show motor function deficits during their first month and deficits increase as they age. C, control (*Chat-Cre; Elp1 or Elp3* ^*+/LoxP*^). *n* = five to seven animals per genotype and sex. Bars = SE. *P* values reach the ≤ 0.05 threshold at five weeks (*Elp1* CKO females and *Elp3* CKO males), six weeks (*Elp1* CKO males), and seven weeks (*Elp3* CKO females) (one-way ANOVA, Turkey’s HSD test). For individual data points and *P* values, see Supplementary Data 1. **e**–**j** 6 weeks. Both control (*Chat-Cre; Elp1*^*+/LoxP*^) and CKO (*Chat-Cre; Elp1*^*LoxP/LoxP*^) mice carry a single copy of *Chat-GFP* for visualization of motor neurons. ELP3 protein levels are reduced in large diameter (≥ 440 μm^2^), *Chat-GFP*-positive neurons of *Elp1 CKO* mice (**h**–**j**) compared to the control (**e**–**g**). Scale bar = 100 μm in (**a**), 25 μm in (**e**–**j**).

Unconditional ablation of Elongator subunits (i.e., ablation in all cell types) is embryonic lethal^25^. Thus, to investigate the specific dependence of motor neurons on Elongator, we combined either a floxed *Elp1* or *Elp3* allele with *Cre* recombinase expression driven by the *Chat* promoter to conditionally ablate Elongator in cholinergic neurons (Fig. S1)^26^. Each of these strains has been previously described^27,28^. To verify the selective expression of *Cre* in cholinergic motor neurons, we crossed *Chat-Cre* mice to a GFP reporter strain that produces GFP following Cre-mediated deletion of a *LoxP*-flanked transcriptional blocker within the coding sequence for GFP (*Rosa mTmG*)^29^. As shown in Fig. 2a, *Chat-Cre; Rosa mTmG* mice show robust production of GFP in large spinal cord motor neurons at E16.5. Although smaller motor neurons can also be cholinergic, GFP was only observed sporadically in smaller cells and at reduced levels compared to large motor neurons (Fig. 2a)^26^.

To produce CKO mice, *Chat-Cre; Elp1*^*+/LoxP*^ and *Elp1*^*LoxP/LoxP*^; Chat-*GFP/GFP* mice were crossed (*Chat-GFP* was included for the visualization of motor neurons^30^). The expected genetic classes, including CKOs (*Chat-Cre; Chat-GFP; Elp1*^*LoxP/LoxP*^), were present at the expected 1:1:1:1 Mendelian ratio (84 *Chat-GFP; Elp1*^*+/LoxP*^*: 85 Chat-GFP; Elp1*^*LoxP/LoxP*^*: 85 Chat-Cre; Chat-GFP; Elp1*^*+/LoxP*^*: 81 Chat-Cre; Chat-GFP; Elp1*^*LoxP/LoxP*^; *P* = 0.9882). Spinal cords from adult CKOs exhibit reduced levels of ELP1 protein (Fig. 2b) and the CKOs also exhibit a robust motor phenotype. Although pups appear normal and exhibit normal activity levels and weigh the same as their control littermates during the first few weeks of life (Fig. 2c), paw grip endurance (PaGE) testing shows that *Elp1* CKO mice begin losing motor strength by four weeks of age (Fig. 2d). This loss progresses during the second and third months of life with the animals becoming moribund between 10 and 14 weeks. *Elp1* CKOs also fail to achieve a normal adult weight and weigh less at nine and 12 weeks compared to their weights at five weeks of age (Fig. 2c). In addition, *Elp1* CKOs exhibit hindlimb clasping and tremors (Supplementary Movie 1), and extreme fasciculations during the later stages of disease (Supplementary Movie 2). We quantified the onset and progression of hindlimb clasping using a previously established rubric^31^. As shown in Fig. S2a, *Elp1* CKOs begin showing signs of hindlimb clasping between 21 and 24 days and progress to a full hindlimb clasping phenotype by 35 days. The CKOs also exhibit obvious muscle atrophy upon autopsy, which was quantified by weighing the tibialis anterior, soleus, and gastrocnemius muscles of *Elp1* CKOs and littermate controls (Fig. S2e). All of these are hallmark symptoms of motor neuron disease, including ALS. Finally, disease severity including fasciculations and weight loss are more severe in males than in females (Fig. 2c) with 90% of male CKOs (40 out of 42) reaching a defined humane endpoint by 10 weeks and 87% of females (32 out of 37) persisting until 12 weeks before reaching a similar humane end point (see “Methods”). Although *Chat-Cre; Elp1*^*LoxP/LoxP*^ mice develop an obvious and progressive loss of motor strength, they do not develop limb paralysis.

All six Elongator subunits contribute to its stability and function^32–34^. To determine whether the levels of ELP3, the catalytic subunit of the Elongator complex, are impacted in *Elp1* CKO mice, we used quantitative IF to analyze ELP3 protein levels in large motor neurons within the spinal cord ventral horn. As shown in Fig. 2e–j and Fig. S2f (bar graph), ELP3 levels are significantly reduced in *Elp1* CKO mice (for source data see Dryad, 10.5061/dryad.x0k6djhvb).

Variants of *Elp3*, rather than *Elp1*, are associated with ALS^22,24^. Although ablation of any of the Elongator subunits cripples the function of the entire Elongator complex^32,34^, we sought to determine whether abrogation of *Elp3* would produce a similar motor phenotype. To this end, we generated cholinergic *Elp3* CKO mice using the same genetic strategy (*Chat-Cre; Elp3*^*+/LoxP*^ × *Elp3*^*LoxP/LoxP*^). Unexpectedly, both *Chat-Cre; Elp3*^*LoxP/LoxP*^ CKOs and *Elp3*^*+/LoxP*^ offspring were only present at an approximate one in 14 ratio (eight *Chat-Cre; Elp3*^*LoxP/LoxP*^ and nine *Elp3*^*+/LoxP*^ offspring out of 111 total offspring). Since both the *Elp3* gene and *Chat* are located on *Mus musculus* chromosome 14, this altered segregation is likely due to linkage, in which case the wild-type *Elp3* allele in the original *Chat-Cre* strain would tend to segregate with the *Cre* recombinase gene and the *Elp3*^*LoxP*^ allele would tend to segregate with the wild-type *Chat* allele. Depending on the degree of linkage*, Chat-Cre; Elp3*^*LoxP*^ and *Elp3*^+^ gametes would be rare, reducing the number of progeny in the *Chat-Cre; Elp3*^*LoxP/LoxP*^ and the *Elp3*^*+/LoxP*^ classes. Having a robust ELP3 antibody for IF we were able to directly verify depletion of ELP3 in cholinergic neurons Fig. S3a–h. Although ELP3 protein appeared completely ablated in most Chat-positive neurons, occasional cells, including those ≥ 440 μm^2^, retained normal levels of ELP3 (Fig. S3i–k). Despite their reduced segregation, *Elp3* CKO mice exhibit a phenotype that is very similar to *Elp1* CKOs, including a progressive loss of motor strength (Fig. 2d), a failure to gain weight (Fig. S3l), hindlimb clasping, and fasciculations.

### *Elp1* CKOs exhibit a loss of alpha motor neurons and progressive denervation of the neuromuscular junction

**Fig. 3:**
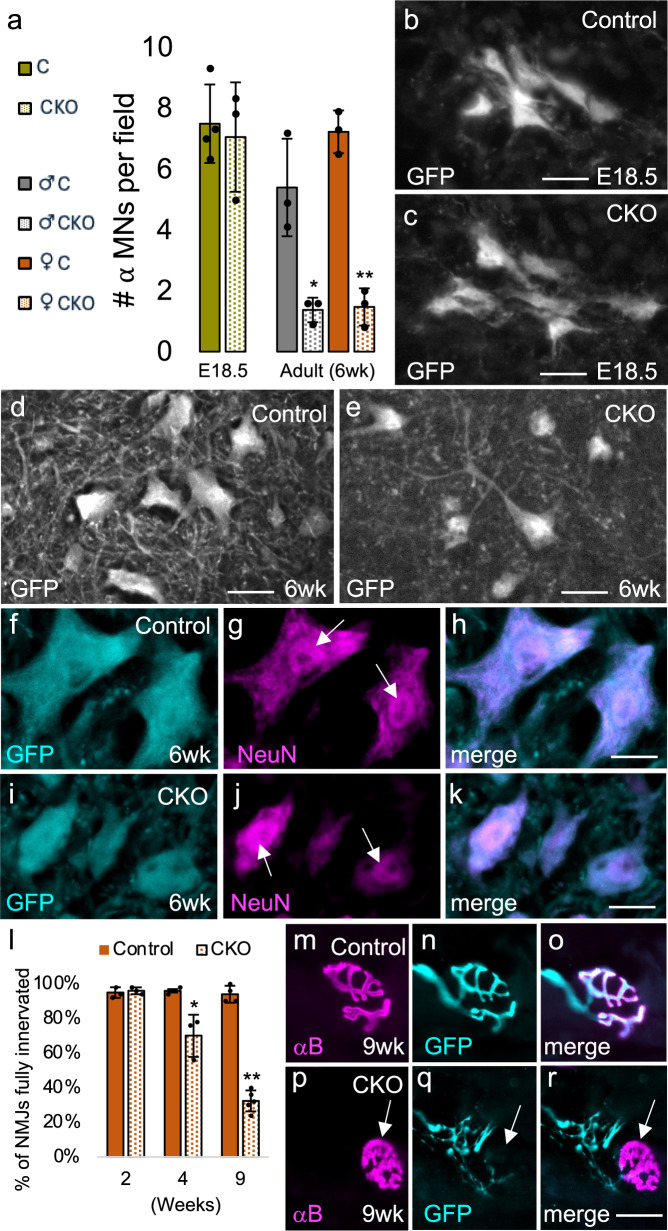
Alpha motor neuron numbers and innervation of the NMJ in *Elp1* CKO mice. **a**–**r** Both control (*Chat-Cre; Elp1*^*+/LoxP*^) and CKO (*Chat-Cre; Elp1*^*LoxP/LoxP*^) mice carry a single copy of *Chat-GFP* for visualization of motor neurons. **a**–**k** Although both genotypes have equal numbers of large (≥ 440 μm^2^), motor neurons at E18.5, the numbers of large, NeuN-positive alpha motor neurons are dramatically diminished by six weeks in both male and female mice. E18.5, *n* = 4 C and 3 CKO; Adult, *n* = 3 male and 3 female mice for each genotype (* *P* < 0.05, ** P < 0.001, unpaired Student’s *t*-test. Bars = SD. See Supplementary Data 2 for source data. **l**–**r** The neuromuscular junction is progressively denervated in CKO mice. *n* = 3 female mice per genotype and time point. *P < 0.05 (4 weeks), **P < 0.001 (9 weeks), unpaired Student’s *t*-test. Bars = SD. **m**–**r** In nine-week-old control mice, motor neuron axons (GFP) completely innervate the NMJ (αB) (**m**–**o**) while in CKO mice (**p**–**r**), the majority are partially to completely denervated (arrow). αB alpha bungarotoxin. Scale bar = 40 μm in (**b**, **c**), 30 μm in (**d**, **e**, **m**–**r**), 15 μm in (**f**–**k**).

To investigate the cause of motor strength loss in *Elp* CKO mice, alpha motor neuron numbers were quantified in *Chat-Cre; Chat-GFP; Elp1*^*LoxP/LoxP*^ mice versus *Chat-Cre; Chat-GFP;Elp1*^*+/LoxP*^ controls at two timepoints: just prior to birth at embryonic day 18.5 (E18.5), and at six weeks of age (Fig. 3a). *Elp1* CKOs were used in this study rather than *Elp3* CKOs because the reduced segregation of *Chat-Cre; Elp3*^*LoxP/LoxP*^ mice was prohibitive in achieving an *n* adequate for quantitative studies. For the embryonic timepoint, alpha motor neurons were identified using three criteria: expression of GFP (*Chat-GFP*-positive), location within the spinal cord ventral horn as shown in Fig. 1c, and a soma area > 440 μm^2^ (less than one percent of gamma motor neurons exceed a soma area of 440 μm^2^)^35,36^. For adult mice, GFP in combination with IF using an anti-NeuN antibody was used to identify alpha motor neurons. Although both alpha and gamma motor neurons are cholinergic (ChAT-positive), the nuclei of alpha motor neurons are NeuN-positive while the nuclei of gamma motor neurons are NeuN-negative. In addition, we also measured the soma area of all GFP-positive, NeuN-positive cells. These data, summarized in Fig. 3, demonstrate that although alpha motor neurons develop normally in *Elp1* CKO mice, the number of large alpha motor neurons (soma area ≥ 440 μm^2^) is drastically reduced by six weeks (Fig. 3a–k). Interestingly, the number of smaller alpha motor neurons (soma area < 440 μm^2^) is elevated in the CKOs (*Chat-Cre; Elp1*^*LoxP/LoxP*^) compared to controls (*Chat-Cre; Elp1*^*+/LoxP*^) (9.2 and 10.0 neurons per field of view in CKO male and female mice respectively, versus 4.9 and 5.1 in control males and females, respectively) (*P* = 0.03, males; <0.001 females) such that the total number of ChAT-positive, NeuN-positive neurons is the same regardless of genotype (8.3 and 10.1 total neurons per field of view in CKO males and females, respectively, versus 9.0 and 10.8 in control males and females respectively) (*P* = 0.58, males; 0.53 females) (see “Methods” for number of mice and sections analyzed, Supplementary Data 2 for source data, and Dryad for images (10.5061/dryad.x0k6djhvb). Innervation of the neuromuscular junction (NMJ) was also quantified at three timepoints (two weeks, four weeks, and nine weeks). Not unexpectedly, given the loss of large motor neuron cell bodies, *Elp1* CKO mice exhibit a progressive loss of innervation that parallels their loss of motor strength (Fig. 3l–r). By nine weeks of age, most NMJs exhibit at least partial denervation with many being completely denervated (arrow in Fig. 3p–r). The above data demonstrate that large alpha motor neurons are selectively lost in *Elp1* CKO mice.

### Both nuclear and nucleolar levels of TDP-43 are diminished in *Elp* CKO mice

Neuronal degeneration in the absence of Elongator has been shown to occur through a variety of pathways, including the unfolded protein response and p53-mediated apoptosis^27,37^. Using a well-established anti-p53 antibody, nuclear p53 was not detected in motor neurons in either controls or *Elp1* CKOs. Using IF, we also examined the levels of CHOP, a transcription factor activated by endoplasmic reticulum stress^38^. Although we did not quantify CHOP fluorescence, its levels in the nucleus of CKO motor neurons appeared normal compared to controls (Fig. S4).

**Fig. 4:**
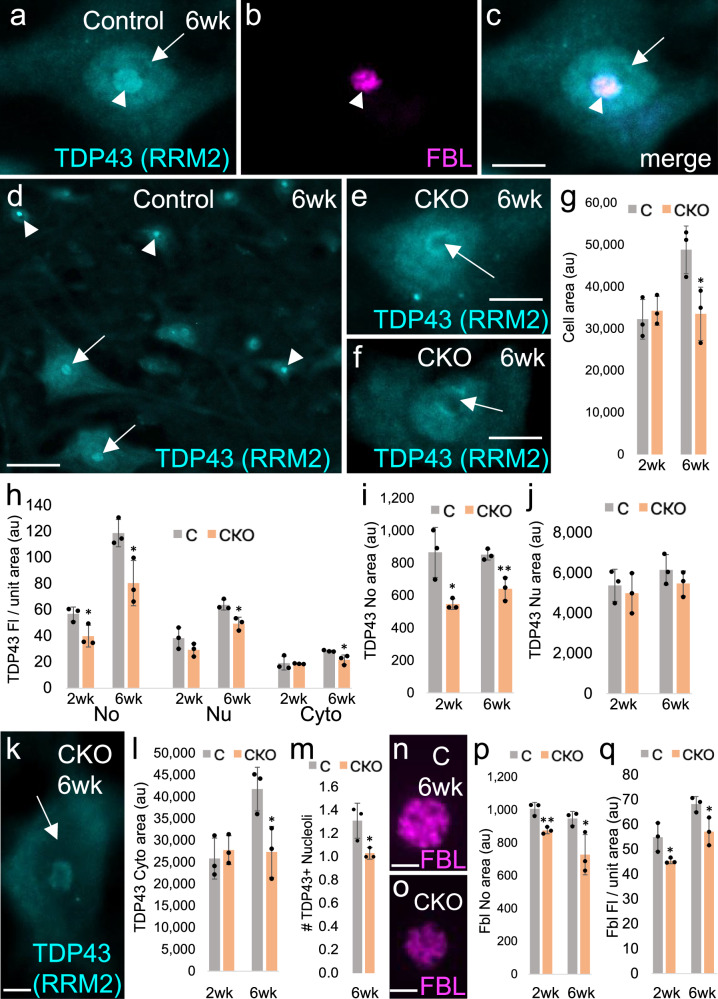
TDP-43 levels are diminished in both the nucleolus and the nucleus of *Elp1* CKO mice*.* All fluorescence quantification was performed using female mice. **a**–**c** IF with anti-TDP-43 antibody 10782-2-AP (Proteintech) that recognizes the second RNA recognition motif (RRM2) shows robust levels of fluorescence in the nucleolus (arrowhead) of neurons and more moderate levels in the nucleus (arrow). **d** Nucleolar TDP-43 is present in large alpha motor neurons (arrows) and in smaller cells, likely interneurons and gamma motor neurons (arrowheads). **e**, **f** Nucleoli of *Elp1* CKO alpha motor neurons exhibit clearing of TDP-43. **g** The average size of large (≥ 440 μm^2^) alpha motor neurons is normal in *Elp1* CKO mice at two weeks of age, but is significantly diminished by six weeks. **h** Quantification of TDP-43 fluorescence per unit area in the nucleolus (No), the nucleus (Nu), and the cytoplasm (Cyto) at two and six weeks in female mice. **i** The nucleolar area occupied by TDP-43 is significantly diminished at both two and six weeks. **j**, **k** The nuclear area occupied by TDP-43 is stable at both two and six weeks, although the fluorescence levels are diminished at six weeks (**h**, **k**). **l** Although normal at two weeks, the cytoplasmic area occupied by TDP-43 is diminished at six weeks. **m** The average number of TDP-43-positive nucleoli per cell is reduced at six weeks of age. **n**–**q** The area occupied by fibrillarin (FBL) (**n**–**p**), and the fibrillarin fluorescence per unit area (**q**) are also reduced in alpha motor neurons of *Elp1* CKO mice. *P < 0.05, **P < 0.01. All *P* values correspond to an unpaired Student’s *t*-test. All bars = SD. For source data, see Supplementary Data 3 (TDP-43), Supplementary Data 4 (FBL), Supplementary Data 5 (# of nucleoli). Au arbitrary units. Scale bar = 6 μm in (**a**–**c**, **e**, **f**, **k**); 36 μm in (**d**); 3 μm in (**n**, **o**).

Cytoplasmic TDP-43-positive aggregates are a hallmark feature of both familial and sporadic ALS. Although it remains unclear as to whether these aggregates are themselves toxic to the cell or instead sequester TDP-43 such that its normal cellular function is compromised, TDP-43 is an established contributor to neurodegeneration in ALS. To determine whether TDP-43 pathology contributes to neuron death in our *Elp1* CKO mouse model, we performed IF with a commonly used, knockout-validated TDP-43 antibody that recognizes the second RNA recognition motif (10782-2-AP, Proteintech)^39^. In optimizing the antibody dilution using control spinal cord sections, we observed a distinct staining pattern with the most prominent fluorescence present in the nucleolus and fainter fluorescence in the surrounding nucleus (Fig. 4a–c). This pattern was consistently observed in almost all large motor neurons, as well as smaller surrounding cells, likely including gamma motor neurons and glia (Fig. 4d), and was not observed in the absence of primary antibody (Fig. S5). Additionally, large motor neurons in *Elp1* CKO mice showed an obvious depletion in nucleolar TDP-43 immunoreactivity with some cells exhibiting partial or almost complete clearing with only a faint rim of fluorescence visible around the nucleolus perimeter (Fig. 4e,f). Questioning a nucleolar function for TDP-43, we surveyed the available literature for documentation of TDP-43’s presence in this organelle. Interestingly, a 2010 study using the same antibody in an immunoelectron-microscopy study of three ALS patients documents similar findings, including the presence of TDP-43 in the nucleolus and its obvious depletion from this organelle in large motor neurons of ALS patients. Since endoplasmic reticulum levels of TDP-43 in these same cells were found to be elevated, the authors attributed the diminished nucleolar levels to mislocalization of TDP-43, rather than to a decrease in its production or longevity. Additionally, a mass spectrometry-based proteomics study investigating the identity and enrichment of nucleolar proteins in the cerebral cortex documents TDP-43’s presence in the nucleolus at a concentration 1.45-fold higher than in the nucleus^40^. To follow up on this previous report, we also compared the concentrations of nucleolar to nuclear TDP-43 via quantitative IF and found a similar level of nucleolar enrichment (1.77-fold ± 0.015, *n* = 3 spinal cords from 6-week adult mice and 25 cells per spinal cord).

Although large (≥ 440 μm^2^) alpha motor neurons are present in *Elp1* CKO mice, they are present in smaller numbers (Fig. 3a). Additionally, by six weeks of age, the average area of motor neurons that meet the 440 μm^2^ cutoff is reduced compared to the control (Fig. 4g). To determine whether the apparent depletion of nucleolar TDP-43 in *Elp1* CKOs might reflect the diminishing size of alpha motor neurons and a proportionate decrease in nucleolar size, rather than a true loss of TDP-43 production or stability, we quantified the level of TDP-43 per unit area in large (≥ 440 μm^2^), CHAT-positive neurons, a primary cell type impacted in ALS. Since our analyses were limited to two fluorescent channels, we used the 440 μm^2^ -size cutoff as a criterion for identifying alpha motor neurons in addition to location within the spinal cord ventral horn (Fig. S6) and expression of *Chat-GFP*. As shown in Fig. 4h, i, the nucleolar TDP-43 fluorescence level per unit area is significantly diminished in large alpha motor neurons in *Elp1* CKOs, as is nucleolar size, at both two and six weeks of age. To determine whether this depletion is due to mislocalization, we also measured TDP-43 fluorescence levels per unit area within the nucleus and the surrounding cytoplasm. Nuclear size is stable in *Elp1* CKO neurons at both two and six weeks (Fig. 4j), and although we did not detect a significant difference in the level of nuclear TDP-43 at two weeks, the trend is in the diminished direction, and by six weeks nuclear TDP-43 is clearly depleted in many large motor neurons (Fig. 4h, k). Similarly, although the area of the cytoplasm and the cytoplasmic TDP-43 fluorescence per unit area are normal at two weeks, both are diminished by six weeks (Fig. 4h, l). Additionally, although the presence of multiple TDP-43-positive nucleoli in a single cell was rare in both the control and the CKO at two weeks of age and was not quantified, by six weeks, *Elp1* CKO motor neurons are less likely to house multiple nucleoli than are controls (Fig. 4m). Finally, we used an anti-fibrillarin antibody to determine whether an impact on the nucleolus in *Elp1* CKO mice is consistent across multiple nucleolar markers. As shown in Fig. 4n–q, both the area of fibrillarin and the fibrillarin fluorescence per unit area are significantly decreased in the CKO at both time points, an indicator of nucleolar stress^41^.

To determine whether *Elp3* CKO mice exhibit similarities in TDP-43 pathology to *Elp1* CKOs, we also quantified the levels of TDP-43 in *Elp3* CKO mice and found similar, but slightly more significant differences in TDP-43 levels. Notably, TDP-43 fluorescence levels per unit area are already significantly reduced by two weeks of age in both the nucleus and the cytoplasm, in addition to the nucleolus (Fig. S7). This difference may reflect the different backgrounds on which the floxed *Elp1* and *Elp3* strains were generated.

Since numerous isoforms of TDP-43 have been documented, including multiple forms that are C-terminally truncated^42–44^, we also tested a knockout validated and commonly used C-terminal TDP-43 antibody. Interestingly, this antibody exhibits a clear absence of immunoreactivity in the nucleolus of motor neurons (Fig. S8a). This result indicates that the nucleolar TDP-43 isoform recognized by the RRM2 antibody does not possess the full-length C-terminus (i.e., that nucleolar TDP-43 is likely a C-terminally truncated isoform).

Given the large body of TDP-43 literature, including multiple IF studies using an N-terminal antibody, we questioned why TDP-43’s presence in the neuron nucleolus is not better represented in the literature. In examining the methods for these previous studies, many include an antigen retrieval step. To determine whether antigen retrieval may interfere with detection of TDP-43 in the nucleolus, we performed IF using the RRM2 antibody both with and without antigen retrieval. Interestingly, the two different protocols give different results. Without antigen retrieval, a prominent TDP-43-positive nucleolus is obvious against the background of a slightly less fluorescent nucleus as shown in Fig. 4a. With antigen retrieval, fluorescence is relatively homogenous throughout the nucleus, with the region corresponding to the nucleolus (fibrillarin-positive) being indistinguishable from the remainder of the nucleus (Fig. S8b–d). We also examined the TDP-43 IF pattern using antigen retrieval on sections from *Elp1* CKOs. In this case, the area corresponding to the nucleolus was often recognizable by an absence of fluorescence or by reduced levels of fluorescence (Fig. S8e–g). To quantify this difference, we measured fluorescence levels of the entire nucleus (including the area corresponding to the nucleolus) in both controls and CKOs, and a significant difference was again detected, likely corresponding to reduced nucleolar levels of TDP-43 in the CKO (Fig. S8h).

## Discussion

Multiple studies associate dysfunction of the Elongator complex with ALS and/or FTD^22–24^. These studies suggest that dysfunction of Elongator may be a common underlying mechanism that contributes to both familial and sporadic forms of the disease. To gain insight into the molecular nature of Elongator’s contribution to ALS, we generated CKO mice in which either *Elp1* or *Elp3* is selectively ablated in cholinergic neurons, including spinal cord alpha motor neurons, the cell type typically impacted first in ALS. The presence of a strong motor phenotype in both *Chat-Cre; Elp1*^*LoxP/LoxP*^ *and Chat-Cre; Elp3*^*LoxP/LoxP*^ mice indicates that Elongator is essential to the survival of motor neurons, and that compromising Elongator function in neurons results in neurodegeneration even in the absence of other mutations. Using this model, we demonstrate that Elongator loss leads to nucleolar shrinkage and depleted levels of both nuclear and nucleolar TDP-43, findings that have not been associated with Elongator in previous studies.

TDP-43 functions in numerous aspects of RNA metabolism, including transcriptional regulation, alternative splicing, and mRNA stabilization and transport^13,14^. Importantly, TDP-43-positive aggregates are present in the vast majority of ALS cases, while mutations in the gene encoding TDP-43 (*TARDBP*) account for only 3% of familial ALS cases and < 1% of sporadic cases^45–47^. Thus, TDP-43 pathology is a key and pervasive feature of the disease irrespective of origin. Full-length TDP-43 is known to autoregulate its protein levels by binding to the 3′ UTR of its own transcript, which results in the splicing of polyadenylation sites that are needed for TDP-43’s exit from the nucleus for translation^42,44^. Interestingly, this autoregulation has been shown to depend on a glycine-rich domain located at the C-terminus^42,44^. All C-terminally truncated isoforms lack this domain^48^, indicating that their levels are likely regulated via a different pathway. Here we show that a C-terminally truncated TDP-43 isoform resides in the motor neuron nucleolus, and that its levels are depleted in the absence of Elongator function. These results indicate that this C-terminally truncated TDP-43 isoform may be regulated, either directly or indirectly, by Elongator.

Although the literature is saturated with articles investigating TDP-43, few document the presence of TDP-43 in the neuron nucleolus^49^. Our data demonstrating that antigen retrieval obscures the visualization of nucleolar TDP-43 may help explain why TDP-43’s presence in this organelle is underrepresented. In spite of this scarcity, a mass spectrometry study of the cerebral cortex documents a 1.45-fold enrichment of TDP-43 in the nucleolus compared to the nucleus^40^. Since nucleoli in this study were collected without exposure to fixative or any antigen retrieval steps that can potentially add artifacts to immunofluorescence protocols, this study provides an important unbiased corroboration of TDP-43’s presence in the neuron nucleolus^40^.

Although alpha motor neurons in *Elp* CKO mice likely suffer from numerous insults in addition to TDP-43 misregulation, our findings raise the question as to whether a critical factor in ALS might be the clearing of TDP-43 from the nucleus and in particular, from the nucleolar subdomain of the nucleus. Recent findings demonstrating that nucleolar dysfunction is a common and key feature of both sporadic and familial ALS, and that this dysfunction is detectable prior to other hallmark ALS cellular pathologies, support this hypothesis^16,17,19–21^. A 2010 immuno-electron microscopy study documenting TDP-43’s depletion from nuclei and particularly from nucleoli of large anterior horn neurons of ALS patients, including in normal-appearing neurons, further supports this possibility. Given the function of nucleoli in ribosome biogenesis, including serving as the site of rRNA transcription and processing, our findings also beg the question as to whether a C-terminally truncated TDP-43 isoform may function in ribosomal RNA processing. In this case, it is possible that nucleolar clearing of TDP-43 could compromise ribosome composition and functioning, potentially leading to translational difficulties and aggregated proteins. Finally, since constitutive ablation of *Elp* subunits is embryonic lethal^25^, our CKO strategy in which *Elp* subunits are selectively ablated in cholinergic neurons, provides a powerful approach for specifically interrogating Elongator-regulated pathways that contribute to alpha motor neuron degeneration. Although not previously understood to be connected, here we have used this model to demonstrate that two well-established cellular pathologies of ALS (TDP-43 dysfunction and nucleolar stress), are likely directly related. Further studies with this model should help determine whether specific TDP-43 isoforms depend on Elongator-mediated tRNA modifications for translational efficiency, as well as identify pathways in addition to TDP-43 that contribute to alpha motor neuron demise in the context of Elongator loss.

## Materials and methods

### Mice

All experiments with animals were performed according to the National Institutes of Health Guide for Care and Use of Laboratory Animals and were approved by the Montana State University Institutional Animal Care and Use Committee under Protocol number 2023-65-1A. As such, we have complied with all relevant ethical regulations for animal use. All mice were maintained in individually ventilated caging systems. Non-recirculated, HEPA-filtered air was provided to the units in a positive mode. Autoclaved pine or sani-chip was used as direct bedding. A commercial diet was fed *ad lib* and mice were provided with chlorinated, reverse osmosis water. At four weeks of age, gel packs were placed on the floor of all cages containing CKOs to ensure hydration without needing to climb. For enrichment, mice were provided with an autoclaved cotton nestlet, autoclaved Enviro-Dri^TM^, plastic shelters, and mice were group housed whenever possible. Cages were opened in a biological safety cabinet. All mice were euthanized via CO_2_ inhalation followed by cervical dislocation as a secondary measure. *Elp1*^*tm1a(KOMP)Wtsi*^ “knockout first” mice containing a frt-flanked *LacZ Elp1* (previously known as *Ikbkap*) reporter that disrupts *Elp1* expression before the *LoxP* flanked 4th exon were obtained from the International Knockout Mouse Consortium (MGI ID: 1914544). This strain was generated on a C57Bl/6N background and has been previously described^27^. Following Flippase-mediated removal of the *LacZ* cassette, the resulting *Elp1*^*LoxP*^ strain (Fig. S1a) was backcrossed onto a C57Bl/6J background more than 10 times. *Elp1* CKO mice (*Chat-Cre; Elp1*^*LoxP/LoxP*^) were generated by crosses between *Chat-Cre; Elp1*^*+/LoxP*^ × *Elp1*^*LoxP/LoxP*^*; Chat-GFP/GFP* mice. A humane endpoint for *Elp1* CKO males was defined by a mass ≤ 55% of their control counterparts (*Chat-Cre; Elp1*^*+/LoxP*^; *Chat-GFP*) combined with a hindlimb clasping score of three and constant fasciculations when moving about the home cage. A humane endpoint for *Elp1* CKO females was defined by a weight ≤ 60% of their control counterparts (*Chat-Cre; Elp1*^*+/LoxP*^; *Chat-GFP*) combined with a hindlimb clasping score of three and constant fasciculations when moving about the home cage. Gel packs were placed on the floor of all cages containing CKOs at four weeks of age to ensure hydration without needing to climb. The following strains were purchased from the Jackson Laboratory: *ROSA*^*mT-mG*^, stock no. 007576^29^; *Chat-Cre*, stock no. 006410^26^, *Chat-GFP*, stock no. 007902^30^. *Elp3*^*LoxP*^ mice were derived from *Elp3*^*tm1.1Tac*^ mice (MGI ID: 5704311) on a C57Bl/6 background^37^. *Elp3* CKO mice were generated through the same genetic strategy as *Elp1* CKO mice (*Chat-Cre; Elp3*^*+/LoxP*^ × *Elp3*^*LoxP/LoxP*^) except that the *Chat-GFP* allele was not incorporated into the *Elp3*^*LoxP/LoxP*^ strain. To visualize cholinergic neurons in *Elp3* CKO mice and controls, an anti-ChAT antibody was used (see below). In all experiments utilizing *Chat-Cre*-positive mice, the *Chat-Cre* allele was hemizygous.

### Randomization

Environmental factors, including lighting, temperature, and humidity, were controlled and consistent for all animals, and animal cages were placed randomly within the rack. For weight, PaGE, and hindlimb clasping studies, the order in which the animals were selected for measurement was randomized using an open-access randomization tool. For all IF studies, a random number generator was used to select control sections and cells. All sections and cells from CKOs were used, negating the need for randomization. For the NMJ analysis, muscle sections and individual NMJs were selected randomly using a random number generator (see below).

### Blinding

All weight, PaGE, and hindlimb clasping analyses were performed blind. L.G. allocated the mice into control and CKO groups for data analysis after the data had been collected. All images of motor neurons were captured blind and measurements were allocated into control and CKO groups by L.G. after all measurements and quantification had been completed.

### Inclusion and exclusion criteria

Genotype and sex were the only selection criteria used and were established a priori. For IF studies, tissue sections that were torn, stretched, or otherwise damaged were excluded.

### Hindlimb clasping scoring

Hindlimb clasping was scored using a previously published scoring system^31^. Briefly, the mouse is suspended by the tail and hindlimb position is observed for 10 s. If hindlimbs are consistently splayed outward, away from the abdomen, the mouse is assigned a score of 0. If one hindlimb is retracted toward the abdomen for more than 50% of the time suspended, it receives a score of 1. If both hindlimbs are partially retracted toward the abdomen for more than 50% of the time suspended, it receives a score of 2. If both hindlimbs are entirely retracted and touching the abdomen for more than 50% of the time suspended, it receives a score of 3.

### PaGE testing

Paw grip endurance (PaGE) testing as a measure of motor function was performed as described in Weydt et al. (2003)^50^, and was modified slightly. Briefly, each mouse was placed on a wire lid from a conventional rodent housing cage; the lid was gently shaken to induce gripping and turned upside down (180°). The latency until the mouse released both hind limbs was measured in seconds. Each mouse was tested five times with an arbitrary maximum of 60 s, and the longest latency to fall or release both hind limbs was recorded.

### Immunofluorescence (IF)

All washing, blocking, secondary antibody, and post-fixation steps were performed at room temperature. All other steps were performed at 4 °C unless stated otherwise. Following fixation, tissues were rinsed in PBS, cryoprotected through a series of sucrose solutions in PBS (15%, 30%), incubated for 2 h in a 1:1 mixture of 30% sucrose and optimal cutting temperature (OCT) compound (Tissue-Tek, Torrance, CA), followed by 2 h in OCT. Tissues were then embedded in OCT and frozen in a dry ice ethanol bath. For immunostaining, slides were bathed in tris-buffered saline (TBS) for 10 min, followed by NGS block (10% normal goat serum, 1% glycine, 0.4% Triton ×-100 in 30 mM Tris, 150 mM NaCl) for 1 h, and overnight incubation in primary antibody (in NGS block). Slides were then rinsed in NGS block, incubated in Alexa Fluor secondary antibody (1:2000 in NGS block) for 1 h, rinsed in 3:1 TBS:NGS block, and mounted in Prolong Antifade Diamond (Invitrogen, La Jolla, CA). Control and experimental embryos were cryosectioned on the same day and sections were incubated in primary antibody on the same day that they were sectioned. For antigen retrieval, slides were incubated in citrate buffer at 95 °C for 10 min following sectioning.

### Antibodies

Primary antibodies included the following: GFP (Abcam, ab13970, 1:1000), ELP1 (Abnova PAB12857, 1:800, knockout validated^27,51^. ELP3 (Proteintech, 17016-1-AP, 1:200, knockout validated here using *Chat-Cre; Elp3*^*LoxP/LoxP*^ mice), NeuN (Proteintech, 26975-1-AP, 1:4000, see the manufacturer’s website for validation details), TDP-43 RRM2 (Proteintech, 10782-2-AP, 2 μg/ml, knockout validated). *Note: although the manufacturer’s website describes this antibody as recognizing the TDP-43 N-terminus, Tsuji et al. (2012)^39^, showed the antibody recognizes amino acids 203–209 of human TDP-43, which corresponds to the second RNA recognition motif. TDP-43 C-terminus (Proteintech, 12892-1-AP, 2 μg/ml, knockout validated), Fibrillarin (Novus Biologicals, NB300-269, 1:500, knockdown validated), ChAT (Millipore Sigma AB144P, 1:100, see the manufacturer’s website for validation details). Secondary antibodies used were Alexa Fluor goat anti-rabbit 488, goat anti-mouse 568, donkey anti-chick 488, donkey anti-goat 568 (Invitrogen, 1:2000).

### Immunohistochemistry

LacZ staining was performed as previously described^52^. Briefly, slides with tissue sections (20 μm) were incubated for three hours in 1 ml of ×-gal solution (1 mg/ml ×-gal, 5 mM potassiμmferricyanide, 5 mM potassiμmforrocyanide, 2 mM MgCl2, 0.25% Triton ×-100 in PBS) at 30 °C. Tissue sections were then fixed in 1 ml of 4% paraformaldehyde for 10 min, followed by three washes in TBS. To combine with IF, tissue sections were then incubated in NGS block followed by the application of primary antibody as above.

### Alpha motor neuron quantification

The *Chat-GFP* allele was included in all *Elp1* CKO experiments where alpha motor neurons were quantified or measured. Only cells in the ventral horn (Fig. S6) that were GFP-positive and NeuN-positive were selected for further analysis^35^. Spinal cords were removed from both control (*Chat-Cre; Elp1*^*+/LoxP*^) and CKO (*Chat-Cre; Elp1*^*LoxP/LoxP*^) animals via hydraulic extrusion^53^. The lumbar enlargement was isolated and fixed for 2.5 h at 4 °C in 4% paraformaldehyde in PBS. Sixty 16 μm sections were trimmed to reach the L5/L4 level. Every other 16 μm section was then collected, filling one slide (approximately 48 sections). IF was performed as described above using anti-GFP and anti-NeuN antibodies, and the field of view (40×) containing the highest number of GFP-positive neurons in the ventral horn (lamina IX, Fig. S6) was photographed. For control mice, sections to be photographed were selected randomly using a random number generator. For the CKO, given the reduced number of large motor neurons, all sections were photographed, and the field of view (40×) was positioned to include the largest possible number of large motor neurons. The number of alpha motor neurons (GFP-positive and NeuN-positive) per ventral horn were counted and their areas measured using ImageJ. Averages were then calculated for the 10 sections with the largest number of alpha motor neurons (GFP-positive, NeuN-positive, and an area ≥ 440 μm^2^) present per section. The same approach was used to quantify the number of GFP-positive, NeuN-positive neurons with an area less than 440 μm^2^. For E18.5, embryos were decapitated and fixed in 4% paraformaldehyde in PBS for 2.25 h. Sections were cut at 16 μm and every other section was collected from the mid to upper lumbar axial level, filling two slides. Every other section was photographed, and the number of alpha motor neurons in a single hemisphere (the hemisphere with the highest number of alpha motor neurons) counted per section and an average calculated for the 12 sections with the largest number of alpha motor neurons present per section.

### Neuromuscular junction analysis

The *Chat-GFP* allele was included in both control (*Chat-Cre; Elp1*^*+/LoxP*^) and CKO (*Chat-Cre; Elp1*^*LoxP/LoxP*^) animals to visualize innervation of the NMJ. Anterior tibialis muscles were dissected from female controls and CKOs and fixed for 20 min in 4% paraformaldehyde in PBS at 4 °C. The muscles were then worked up for cryosectioning as described above for spinal cords. Sections were cut at a thickness of 25 μm and every other section collected, filling two to three slides for a total of 60 sections. IF was performed as described above using an anti-GFP antibody to visualize innervation of the NMJ. Alpha bungarotoxin 555 (Molecular Probes, #B160; 1 μg/ml) was included at the secondary antibody step. Sections to be analyzed were selected randomly using a random number generator. NMJs were also randomly selected by generating a number between 1 and 10 and counting to that NMJ starting from the top right corner of the section. 50 NMJs were analyzed per animal and tallied as being either fully innervated (GFP pattern matches the pattern of alpha bungarotoxin), or not fully innervated (GFP either partially or totally missing). Anterior tibialis muscles from three control and three experimental animals were analyzed per time point.

### TDP-43 and Fibrillarin corrected fluorescence and area analysis

Nu area in Fig. 4j corresponds to the entire nucleus (including the nucleolus). Fluorescence levels per unit area were calculated as follows: No = (No corrected total fluorescence (CTF)/No area); Nu = (Nu CTF–No CTF)/(Nu area–No area); Cytoplasm = (total cell CTF–Nu CTF)/(total cell area–Nu area). For TDP-43 and fibrillarin fluorescence measurements, spinal cords from control (*Chat-Cre; Elp1*^*+/LoxP*^) and CKO (*Chat-Cre; Elp1*^*LoxP/LoxP*^) were collected, processed for cryosectioning, and sectioned as described above (alpha motor neuron quantification). IF was performed as described above using either anti-TDP-43 and anti-GFP primary antibodies, or anti-fibrillarin and anti-GFP antibodies. Antigen retrieval was included for fibrillarin. For controls, a random number generator was used to randomly select sections. The field of view in the spinal cord ventral horn (lamina IX, Fig. S6) (40×) with the highest density of GFP-positive neurons was photographed. For CKOs, given the reduced number of surviving motor neurons, all GFP+ cells in the ventral horn were photographed. Identical exposure, gain, and offset settings were used for both control and experimental images. Next, for both controls and CKOs, ImageJ was used to measure the area of photographed, GFP+ cells. In cells with a soma area ≥ 440 μm^2^ and that contained a whole intact nucleus via DAPI staining, TDP-43 or fibrillarin areas and fluorescence levels were measured using Image J processing and analysis software (https://imagej.nih.gov/ij/). A minimum of 20 cells were analyzed per spinal cord for controls and between 8 and 20 for the CKOs given the reduced number of alpha motor neurons with soma areas ≥ 440 μm^2^ in the CKO spinal cords. A minimum of three spinal cords were analyzed per genotype and the exact number of spinal cords for each analysis is shown on the individual bar graphs in Fig. 4.

### Statistics and reproducibility

Statistical analyses were performed using Excel, Prism, example and open-access one-way ANOVA and Tukey HSD software. Data are presented as mean ± standard deviation (SD), unless stated otherwise. For comparisons between two groups, a two-tailed unpaired Student’s *t*-test was used. For multiple group comparisons, one-way ANOVA was applied, followed by Tukey’s post hoc test.

Sample sizes were based on pilot studies and were calculated using a statistical power of 80% and a significance level of 0.05. Randomization was employed in all studies except in the selection of microscopic fields of view and large diameter (≥ 440 μm^2^) alpha motor neurons for CKOs. Since the number of large diameter alpha motor neurons is drastically reduced in *Elp* CKO mice, all lumbar enlargement ventral horn fields of view were captured and analyzed and all large diameter alpha motor neurons were included in our analyses. All experiments were independently replicated at least three times to ensure reproducibility and the number of biological replicates (*n*) are indicated in the figure legends and/or methods. All raw source data are available in the Supplementary Data files, or at Dryad (10.5061/dryad.x0k6djhvb).

### Microscopy

Images were captured using a Nikon TE200 inverted microscope equipped with a QImaging QICAM 12-bit Mono Fast 1394 Cooled camera and SPOT software. Identical exposure times, gain, and offset settings were used to capture control and experimental images.

### Reporting summary

Further information on research design is available in the Nature Portfolio Reporting Summary linked to this article.

## Supplementary information

Supplementary Information

Description of Additional Supplementary Files

Supplementary Dataset 1

Supplementary Dataset 2

Supplementary Dataset 3

Supplementary Dataset 4

Supplementary Dataset 5

Supplementary Movie 1

Supplementary Movie 2

Reporting summary

## Data Availability

Source data for all charts and graphs are available in Supplementary Data files 1–5 or at Dryad (10.5061/dryad.x0k6djhvb). Photomicrographs used to generate source data are also available at Dryad. All other data are available from the corresponding author upon reasonable request.
