# Supplementary material for: An Elongator mouse model of ALS spotlights TDP-43 in the motor neuron nucleolus
Source: Commun Biol. 2025 Aug 21;8:1259. doi: 10.1038/s42003-025-08701-9 (PMC12370970; doi:10.1038/s42003-025-08701-9)
Supplement: Supplementary file 1 — Supplementary Information [file 42003_2025_8701_MOESM1_ESM.pdf]

Supplementary Information

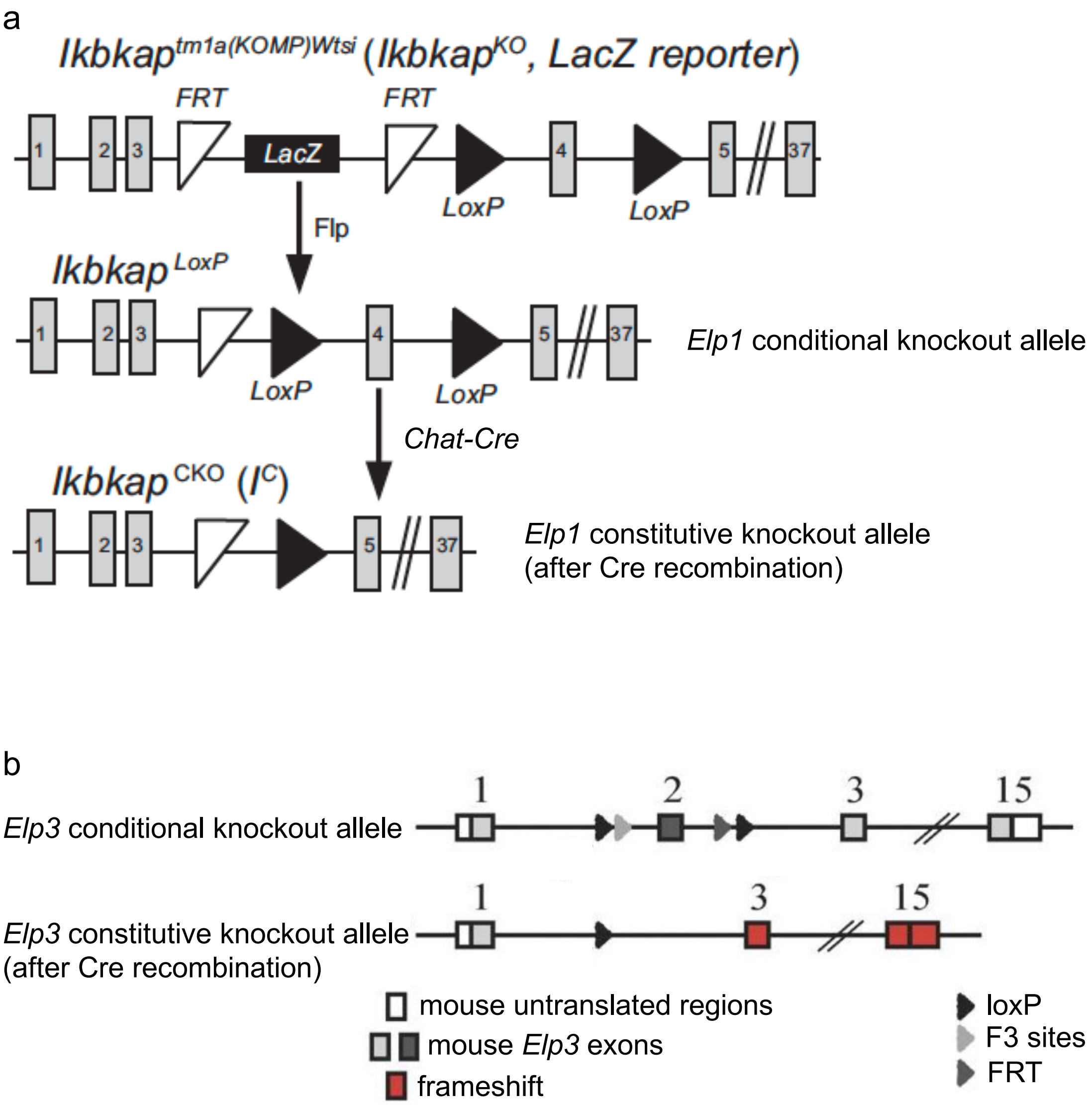

Figure S1. ***Elp* conditional knockout mice.** a) Schematic illustrating the *Elp1* (previously known as *Ikbkap*) CKO strategy as previously published[27]. Schematic illustrating the *Elp3* CKO strategy as previously published[28].

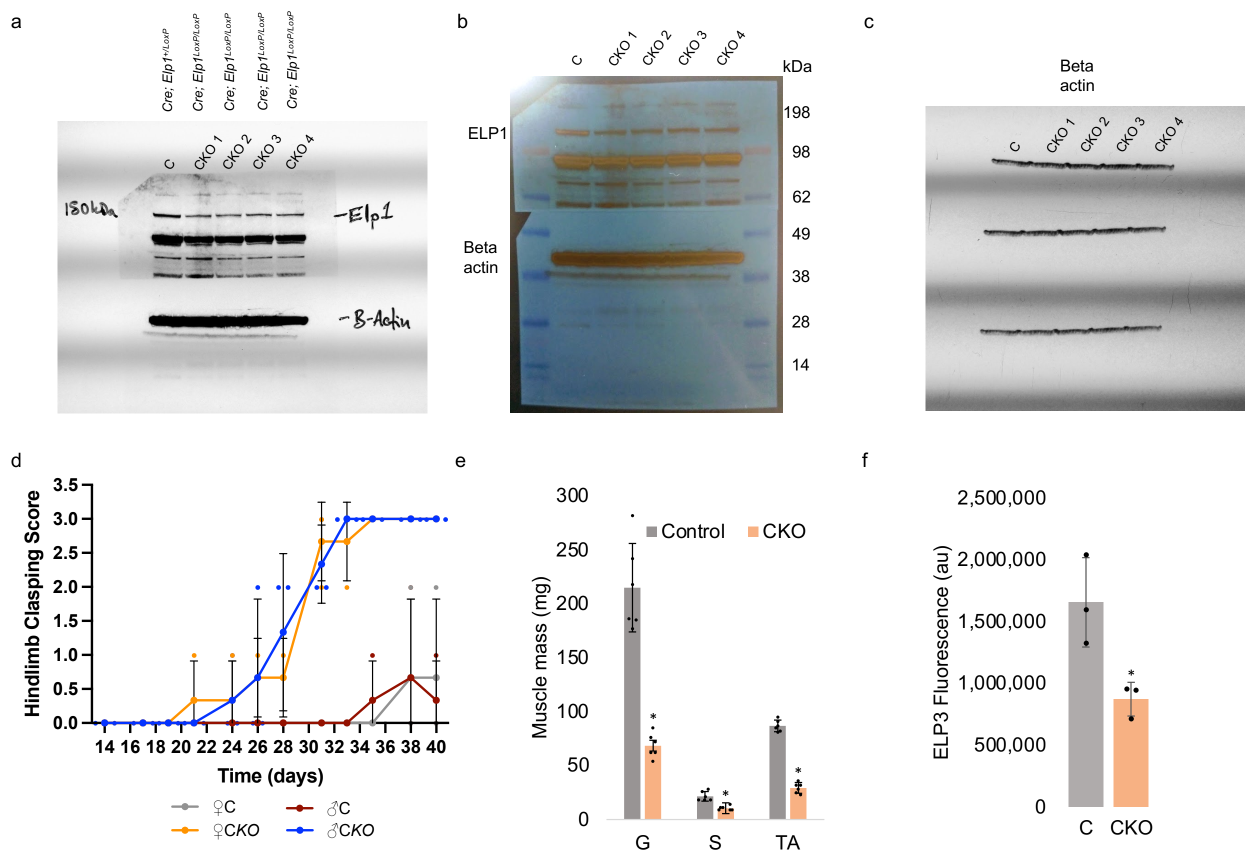

Figure S2. ***Elp1* CKO mice exhibit additional hallmarks of neurodegeneration and diminished levels of ELP3.** a-c) ELP1 western blot. After blotting, the membrane was cut at approximately 60 kDa so that each half of the blot could be probed separately for either ELP1 or beta actin. a) Uncropped image of western blot film shown in Fig. 2b (20 second exposure). b) Western blot film shown in Figs. 2b and S2a overlaid on the western blot membrane with size standards. c) Uncropped image of the beta actin blot shown in Figs. 2b and S2a. The image shows three different exposures. With each exposure, the membrane was briefly applied to the film (less than 1 second), and then moved to a lower position on the film in hopes of achieving adequate band resolution without overexposure. d-f) Control, C = *Chat-Cre; Elp1*$^{+/LoxP}$; conditional knockout, CKO = *Chat-Cre; Elp1*$^{LoxP/LoxP}$. d) Hindlimb clasping. Male and female *Elp1* CKO mice begin exhibiting signs of hindlimb clasping at 21-24 days and progress to a full hindlimb clasping phenotype (Score of 3) between 33 and 35 days. *n* = three mice for each sex and genotype. *P* values become significant at 31 days for both sexes ($P < 0.01$, unpaired Student's t-test). For individual *P* values, see Dryad (https://doi.org/10.5061/dryad.x0k6djhvb). e) Muscle wasting. Masses of the gastrocnemius,

soleus, and tibialis anterior in male control and *Elp1* CKO mice at 10 weeks of age. *n* = six samples per muscle type and genotype. * $P < 0.001$, unpaired Student's t-test. f) ELP3 levels are significantly depleted in *Elp1* CKO mice. * $P = 0.02$, unpaired Student's t-test. All bars = SD. For source data, see Dryad (https://doi.org/10.5061/dryad.x0k6djhvb). G, gastrocnemius; S, soleus; TA, tibialis anterior.

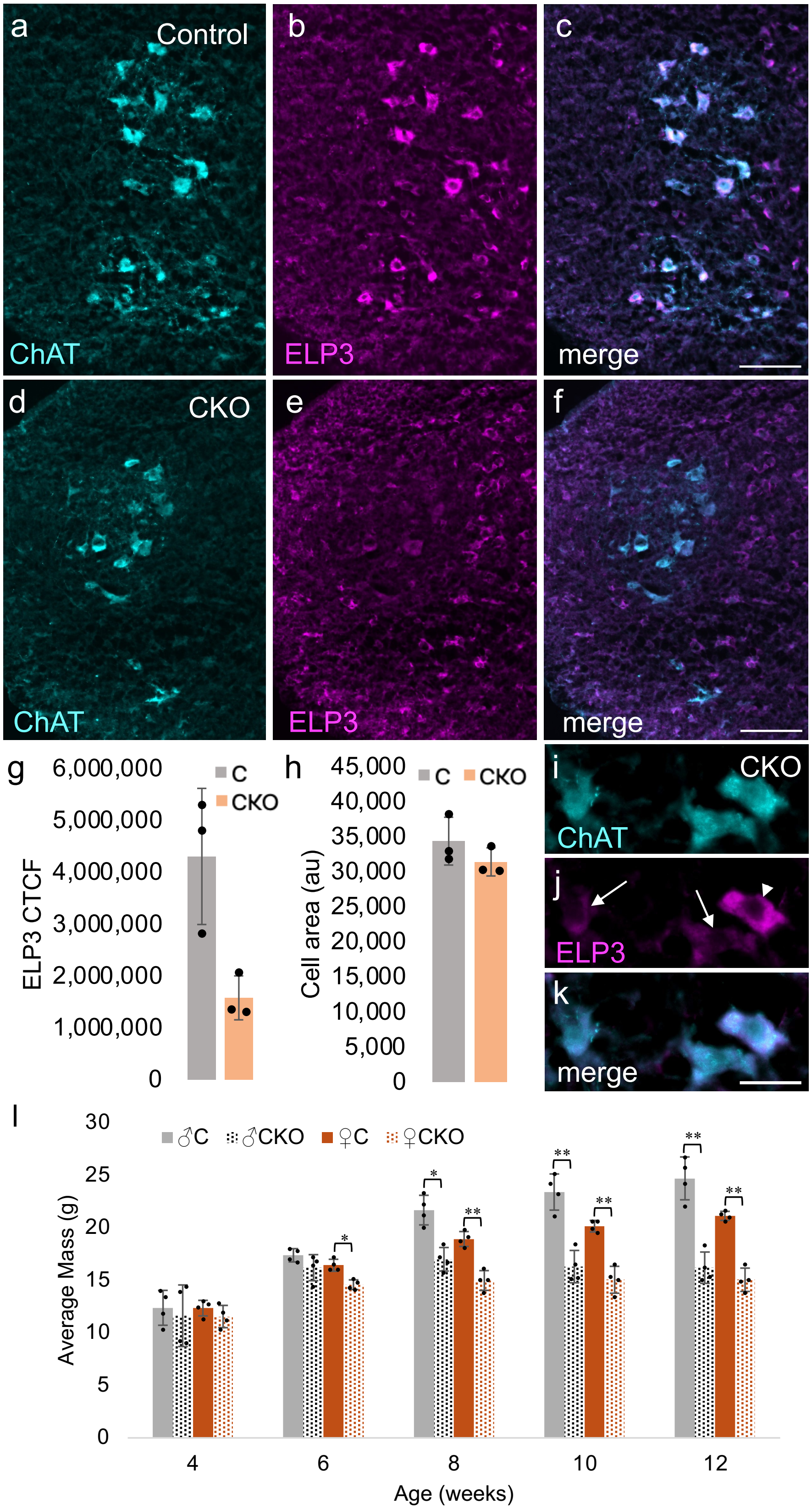

Figure S3. ***Elp3*** **CKO mice.** a-k) *Elp3* CKO mice at two weeks of age. a-g) Elp3 protein levels are severely depleted in most ChAT-positive neurons in the spinal cord ventral horn. g) Quantification of ELP3 fluorescence levels in large (≥ 440 µm2) ventral horn, ChAT-positive neurons. See Dryad.com for source data. h) Similar to *Elp1* CKO mice at two weeks of age, the area of large, ChAT-positive neurons is the same as in control mice. i-k) Although ELP3 protein is completely ablated in most ChAT-positive neurons (arrows), some cells retain normal levels of ELP3 (arrowhead). l) *Elp3* CKO mice fail to achieve a normal adult weight, with their maximum weight occurring between six and eight weeks, similar to *Elp1* CKO mice. **P* < 0.01, ***P* < 0.001, unpaired Student's t-test. Bars = SD. For source data, see Supplementary Data 1. Scale bar = 100 µm in a-f, 25 µm in i-k. C, Control (*Chat-Cre; Elp3*$^{+/LoxP}$); CKO, conditional knockout (*Chat-Cre; Elp3*$^{LoxP/LoxP}$).

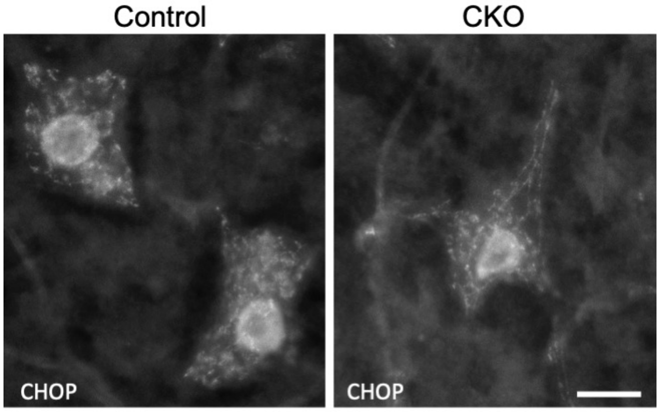

Figure S4. CHOP levels appear normal in alpha motor neurons of *Elp1 CKO* mice. Representative images of CHOP in alpha motor neurons in the lumbar enlargement from control and CKO mice at six weeks of age. Control (*Chat-Cre; Elp1*$^{+/LoxP}$); CKO (*Chat-Cre; Elp1*$^{LoxP/LoxP}$). Scale bar = 12 um.

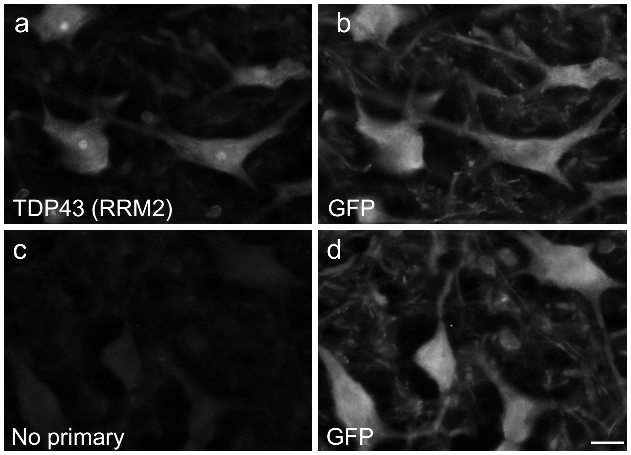

Figure S5. No primary antibody control. a-d) Sections from the lumbar enlargement of a 6-week adult female carrying the *Chat-GFP* allele. a) IF using anti-TDP-43 RRM2 antibody and goat anti-rabbit secondary. b) IF for GFP on the same section as shown in a. c) IF in which the anti-TDP-43 antibody was omitted, but goat anti-rabbit secondary was applied. d) IF for GFP on the same section as shown in c. Scale bar = 10 μm.

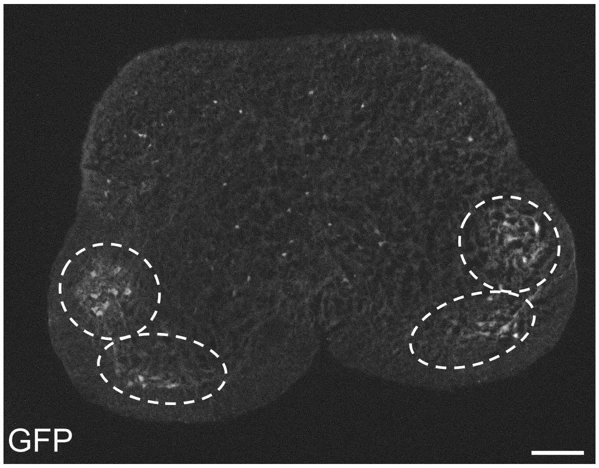

Figure S6. Spinal cord ventral horn motor neurons. Representative image of a cross section through the lumbar enlargement of the spinal cord in a two-week-old mouse carrying the *Chat-GFP* allele. Dashed circles show the motor neuron pools in the spinal cord ventral horn that were used in all studies. Scale bar = 150 µm.

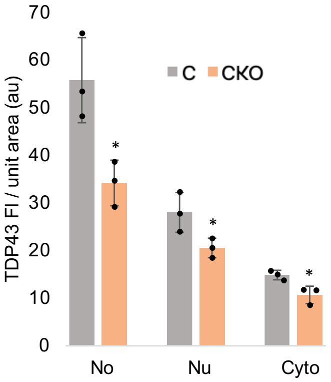

Figure S7. TDP-43 levels are diminished in *Chat-Cre; Elp3$^{LoxP/LoxP}$* CKO mice. TDP-43 fluorescence levels were quantified at 2 weeks of age. *$P < 0.05$, unpaired student's t-test. Bars = SD. C, Control (*Chat-Cre; Elp3$^{+/LoxP}$*); CKO, conditional knockout (*Chat-Cre; Elp3$^{LoxP/LoxP}$*); No, nucleolus; Nu, nucleus; Cyto, cytoplasm.

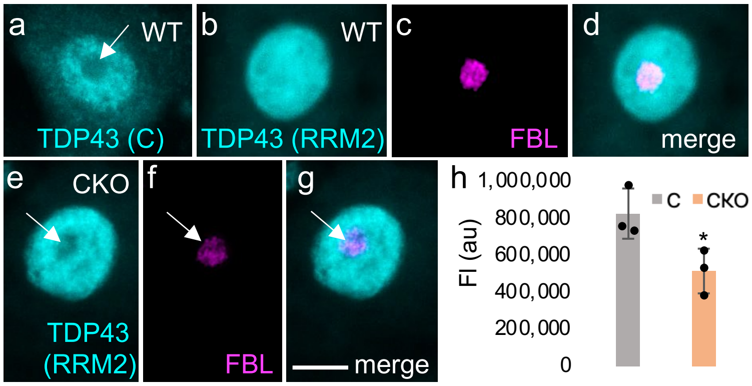

Figure S8. TDP-43 Immunofluorescence. a-g) Cross sections through the lumbar enlargement showing alpha motor neurons in the ventral horn of female wild-type mice (a-d) and *Elp1* CKO mice (e-g) at six weeks of age. a) A clear absence of nucleolar immunoreactivity is observed when IF is performed using a C-terminal TDP-43 antibody. b-d) When antigen retrieval is performed in combination with IF using the TDP-43 RRM2 antibody, the nucleolus is no longer distinguishable from the surrounding nucleus. e-g) Diminished nucleolar immunoreactivity (arrow) is observed in alpha motor neurons of *Elp1* CKO mice when IF is performed using the RRM2 TDP-43 antibody in combination with antigen retrieval. h) Quantification of nuclear TDP-43 immunoreactivity using the RRM2 antibody in combination with antigen retrieval shows diminished levels in *Elp1* CKO (*Chat-Cre; Elp1*$^{LoxP/LoxP}$) mice compared to the control (*Chat-Cre; Elp1*$^{+/LoxP}$). See Supplementary Data 5 for source data. WT, wild-type; RRM2, RNA recognition motif two. FBL, fibrillarin. $*P < 0.01$, unpaired Student's t-test. Bar = 100 µm in a-f, 40 µm in i-k.
