# Supplementary material for: An Elongator mouse model of ALS spotlights TDP-43 in the motor neuron nucleolus
Source: Commun Biol. 2025 Aug 21;8:1259. doi: 10.1038/s42003-025-08701-9 (PMC12370970; doi:10.1038/s42003-025-08701-9)
Supplement: Supplementary file 2 — Description of Additional Supplementary Files [file 42003_2025_8701_MOESM2_ESM.docx]

**Description of Additional Supplementary Files**

Supplementary Data 1: Source data for mass and PaGE quantification

Supplementary Data 2: Source data for motor neuron counts

Supplementary Data 3: Source data for TDP43 quantification

Supplementary Data 4: Source data for fibrillarin quantification

Supplementary Data 5: Source data for nucleoli number quantification and TDP43 quantification with antigen retrieval

Supplementary Movie 1: Video showing hindlimb clasping and fasciculations in Elp1 CKO mice

Supplementary Movie 2: Video showing tremors in Elp1 CKO mice
